# Supplementary material for: Organic electrochemical transistor arrays for real-time mapping of evoked neurotransmitter release in vivo
Source: eLife. 2020 Feb 11;9:e50345. doi: 10.7554/eLife.50345 (PMC7075691; doi:10.7554/eLife.50345)
Supplement: Transparent reporting form [file elife-50345-transrepform.docx]

***eLife’s* transparent reporting form**

We encourage authors to provide detailed information *within their submission* to facilitate the interpretation and replication of experiments. Authors can upload supporting documentation to indicate the use of appropriate reporting guidelines for health-related research (see [EQUATOR Network](http://www.equator-network.org/%20)), life science research (see the [BioSharing Information Resource](https://biosharing.org/)), or the [ARRIVE guidelines](http://www.plosbiology.org/article/info:doi/10.1371/journal.pbio.1000412) for reporting work involving animal research. Where applicable, authors should refer to any relevant reporting standards documents in this form.

If you have any questions, please consult our Journal Policies and/or contact us: [editorial@elifesciences.org](mailto:editorial@elifesciences.org).

**Sample-size estimation**

- You should state whether an appropriate sample size was computed when the study was being designed
- You should state the statistical method of sample size computation and any required assumptions
- If no explicit power analysis was used, you should describe how you decided what sample (replicate) size (number) to use

Please outline where this information can be found within the submission (e.g., sections or figure legends), or explain why this information doesn’t apply to your submission:

The sample size of each statistical analysis is states as ‘n = x’ in the caption below the figure. Specifically, x is for number of repeated measurements (trial) using the device or animals (biological replication) involved.

For Figure 2d, n = 3 trials.

For Figure 3d, the number next to each box states the number of trials. The parameters for each box are stated in the caption.

For Figure 3e, n = 3 rats. At least 20 trials were obtained from each rat.

For Figure 4c, n = 34 trials (also stated in the caption).

For Figure 4d&e, n = 3 rats. ~30 trials were obtained from each rat.

For Figure 4f, n = 8 rats. ~30 trials were obtained from each rat.

For Figure 5c, n = 10 rats. ~30 trials were obtained from each rat.

For Figure 6c-e, n = 6 rats. ~30 trials were obtained from each rat.

**Replicates**

- You should report how often each experiment was performed
- You should include a definition of biological versus technical replication
- The data obtained should be provided and sufficient information should be provided to indicate the number of independent biological and/or technical replicates
- If you encountered any outliers, you should describe how these were handled
- Criteria for exclusion/inclusion of data should be clearly stated
- High-throughput sequence data should be uploaded before submission, with a private link for reviewers provided (these are available from both GEO and ArrayExpress)

Please outline where this information can be found within the submission (e.g., sections or figure legends), or explain why this information doesn’t apply to your submission:

The experiment was normally performed twice per week.

One independent rat was defined as a biological replication. Please refer to captions of corresponding figures or the ‘Sample-size estimation’ above.

**Statistical reporting**

- Statistical analysis methods should be described and justified
- Raw data should be presented in figures whenever informative to do so (typically when N per group is less than 10)
- For each experiment, you should identify the statistical tests used, exact values of N, definitions of center, methods of multiple test correction, and dispersion and precision measures (e.g., mean, median, SD, SEM, confidence intervals; and, for the major substantive results, a measure of effect size (e.g., Pearson's r, Cohen's d)
- Report exact p-values wherever possible alongside the summary statistics and 95% confidence intervals. These should be reported for all key questions and not only when the p-value is less than 0.05.

Please outline where this information can be found within the submission (e.g., sections or figure legends), or explain why this information doesn’t apply to your submission:

All the statistical analysis was carried out using students’ two-tailed ttest.

For each boxplot (Figure 3d), the whisker range and percentile of each box were stated in the caption.

For other statistical analysis, mean values were used and the error bars were standard error.

For each fitting, the Pearson’s R value was stated in the corresponding figure.

(For large datasets, or papers with a very large number of statistical tests, you may upload a single table file with tests, Ns, etc., with reference to sections in the manuscript.)

**Group allocation**

- Indicate how samples were allocated into experimental groups (in the case of clinical studies, please specify allocation to treatment method); if randomization was used, please also state if restricted randomization was applied
- Indicate if masking was used during group allocation, data collection and/or data analysis

Please outline where this information can be found within the submission (e.g., sections or figure legends), or explain why this information doesn’t apply to your submission:

This information does not apply to this submission.

**Additional data files (“source data”)**

- We encourage you to upload relevant additional data files, such as numerical data that are represented as a graph in a figure, or as a summary table
- Where provided, these should be in the most useful format, and they can be uploaded as “Source data” files linked to a main figure or table
- Include model definition files including the full list of parameters used
- Include code used for data analysis (e.g., R, MatLab)
- Avoid stating that data files are “available upon request”

Please indicate the figures or tables for which source data files have been provided:

Main Matlab code and sample data set (raw csv files from sourcemeter) to convert ISD to delta Vg-eff are provided. The sample output (xlsx file) is also provided.
